# Steroidomics of Pregnant Women at Advanced Age

**DOI:** 10.3389/fendo.2022.796909

**Published:** 2022-02-23

**Authors:** Bin Yu, Fang Guo, Yuqi Yang, Wei Long, Jun Zhou

**Affiliations:** Department of Medical Genetics, Changzhou Maternal and Child Health Care Hospital, Changzhou, China

**Keywords:** advanced maternal age, steroid, hormone, pregnancy complication, placenta, mass spectrometry

## Abstract

**Objectives:**

To discover the profiles of different steroid hormones at the maternal-fetal interface and reveal the change characteristics in pregnant women at advanced maternal age (AMA).

**Methods:**

Forty pregnant women were recruited in the study, including 20 AMA women (age ≥ 35) and 20 normal controls (age < 35 and without pregnancy complications). Among AMA women, 6 (AMA2) had pregnancy complications, and 14 (AMA1) had no complications. Their maternal blood (MB), placental tissue (P), and fetal cord blood (CB) were collected, and 18 different steroid hormone metabolites were analyzed by liquid chromatography tandem mass spectrometry (LC-MS/MS).

**Results:**

The estradiol (E2) levels in MB were higher than those in P and CB. In contrast, the estrone (E1) and estriol (E3) levels were higher in P and CB. Compared with the progesterone levels (P4) in MB, those in P and CB were higher; however, cortisol (F) levels were deficient. In contrast, F in MB was maintained at an elevated level. Further, cortisone (E) levels in CB were higher than those in MB and P. Except for the decline of testosterone (T), androstenedione (A2) and Dihydrotestosterone (DHT), there were no significant differences in the other 15 steroid hormones in MB between the AMA1 and the control group (p>0.05). Compared with the AMA1 group, androgen levels were significantly higher in AMA2, especially in T (1.55 *vs.* 0.68 ng/ml, p=0.023), A2 (2.27 *vs.* 0.92 ng/ml, p=0.011) and Dehydroepiandrosterone (DHEA) (2.39 *vs.* 1.50 ng/ml, p=0.028). However, there were no significant changes in P and CB between two groups.

**Conclusion:**

There are distribution rules and cascade changes of steroid profiles in maternal-fetal compartments. Significantly high androgen levels in AMA women have a positive relationship with adverse pregnancy complications.

## Introduction

Women at advanced maternal age (AMA) are at high risk of poor maternal and neonatal outcomes, including the babies with congenital disabilities, but also preeclampsia (PE), gestational diabetes mellitus, premature rupture of membranes, and others (1, 2). However, the mechanisms of increased pregnancy risk caused by a pregnant woman’s age remain unclear.

As a complex, dynamic, and heterogeneous organ, the placenta performs gas exchange, nutrient absorption, immune functions, hormone exchange and metabolism between the mother and fetus. Normal placental function is essential for maintaining pregnancy and fetal development. Recently, some studies showed that placental defects might relate to the occurrence and development of adverse pregnancy in AMA women (3). However, the exact molecular mechanism remained elusive. At the same time, the placenta is also an essential endocrine organ during pregnancy as it regulates hormone secretion and mediates hormone exchange between the mother and the fetus (4, 5). The following points are clear: the placenta is the main source of progesterone (P4) and estrogen beyond 9-10 weeks of gestation. Moreover, as an intermediary substrate, testosterone (T) can ultimately be converted to estrogen [estrone (E1), estradiol (E2), and estriol (E3)] by the dehydroepiandrosterone sulfate in the placenta (6). Maternal glucocorticoids can diffuse freely across the placenta and fetal circulation (7). However, the network of hormone synthesis, transmission, and regulation between the mother and fetus is complex, and its role in some adverse pregnancy diseases is still unclear. Additionally, it has not been reported whether the hormones of AMA pregnant women have unique characteristics or are related to the increased pregnancy risk.

To discover the profiles of different steroid hormones at the maternal-fetal interface and reveal the change characteristics in pregnant women at AMA, we analyzed the levels of 18 different steroid hormone metabolites using liquid chromatography tandem mass spectrometry (LC-MS/MS) in maternal blood (MB), placenta tissues (P), and fetal cord blood (CB) simultaneously. We compared the relationship between the diverse types of hormones at the maternal-fetal interface and revealed the change in characteristics of pregnant AMA women. We hope to contribute in explaining the reasons for the high pregnancy risk of AMA women.

## Materials and Methods

### Samples Collection

From April to August 2021, a total of 40 pregnant women who were hospitalized and delivered in Changzhou maternal and child health hospital were recruited in the study, including 20 women at advanced maternal age (AMA group, age ≥ 35) and 20 women as a control group (age < 35, without pregnancy complications, same period). Patients were not recruited consecutively and the choice for inclusion was haphazard. Their clinical characteristics are shown in **Table 1**. Among AMA women, 6 (AMA2) had adverse pregnancy complications, mainly PE and gestational diabetes mellitus, and 14 (AMA1) were AMA pregnant women without pregnancy complications.

**Table 1:** Population characteristics of present study.

	Control	AMA		
		Total	AMA1	AMA2
Number	20	20	14	6
Age (year)	30.50 (28.75-32.00)	36.00 (35.00-39.00)	36.00 (36.00-39.00)	35.50 (35.00-36.75)
Gestational week (weeks)	38.60 (38.35-39.18)	38.60 (38.27-39.10)	38.80 (38.23-39.08)	38.60 (38.38-39.05)
Height (cm)	160.50 (158.00-163.50)	160.00 (157.50-162.00)	159.00 (155.75-162.00)	160.00 (158.50-160.00)
Weight (kg)	70.65 (64.00-78.62)	67.75 (63.00-73.30)	67.25 (63.25-70.38)	78.75 (66.25-82.25)
Weight increase (kg)	15.00 (14.00-16.12)	15.00 (12.15-17.00)	15.00 (12.62-17.75)	15.00 (10.00-15.00)
BMI	26.85 (26.08-28.96)	25.79 (24.95-27.55)	25.75 (24.99-27.35)	29.07 (25.03-32.74)
Newborn Sex (male,%)	9 (45.00%)	9 (45.00%)	7 (50.00%)	2 (33.33%)
Birth weight (g)	3305.00 (3182.50-3782.50)	3430.00 (3230.00-3622.50)	3350.00 (3237.50-3565.00)	3555.00 (3297.50-3812.50)

AMA1, without complications of pregnancy; AMA2, with complications of pregnancy.

After informed consent, their MB, P, and CB were collected. Their MB were collected before delivery, while their P and CB were obtained within 20 min after delivery. MB and CB were allowed to clot at room temperature for one hour, centrifuged at 2000 g for 10 min, and the upper serum samples were stored at -80°C until they were assayed. A small piece of P (approximately 1 cm^3^) from a region 5 cm away and 2 cm deep from the umbilical cord insertion was obtained. The deciduas of the placenta were removed to avoid contamination with the maternal tissue. After removing the basal and the chorial plates, the remaining villous tissue was washed, by phosphate-buffered saline, to clean the MB, which was snap-frozen in liquid nitrogen for 1 min and stored at -80°C until they were assayed. The whole placentas were sent to the pathology department within half an hour, and a professional pathologist dissected and extracted the materials.

### Tissue Extraction

The placenta is taken out from the refrigerator at -80°C, then ground into powder through a high-throughput tissue grinder (Shanghai TEBO Biotechnology and Technology, China). The powder was then cooled in liquid nitrogen to minimize the effect of tissue degradation. Furthermore, the powder was accurately weighed to 0.5 mg and put in a 2 mL centrifuge tube. Next, 500 μL of an 80% methanol aqueous solution was added and mixed before being placed in an ice bath for 5 min. After that, it was centrifuged at 15000 rpm and 4°C for 15 min. Subsequently, 400 μL of the supernatant solution was taken out, and 200 μL of water was added. Lastly, it was centrifuged at 15000 rpm and 4°C for 20 min, and 500 μL of the supernatant solution was taken out to be stored at -80°C for detection.

### Sample Processing

Two hundred microliter of methanol was added to activate the solid-phase extraction (SPE) plate; SPE made the methanol flow out. After that, 200 μL of water was added to moisten the SPE plate, which also made the water flow out through SPE. Next, 200 μL of plasma, the extracted supernatant solution of the placenta, was transferred to a 2 mL centrifuge tube, and 20 μL of internal standard and 300 μL of methanol were added. Subsequently, the tube underwent vortex oscillation for 1 min, and then, 400 μL of water was added before undergoing another 1 min vortex oscillation. Afterward, it was centrifuged at 14000 rpm and 4°C for 5 min.

Furthermore, 700 μL of supernatant solution was taken out to pass through the SPE plate and flow out through SPE. The SPE plate was then washed with 200 μL of a 10% acetonitrile solution, 200 μL of an n-hexane solution, and 40 μL of a 90% acetonitrile solution sequentially, which also flowed out through SPE. After that, the final solution, which flowed out from the SPE with a 96-well plate, was collected, and 60 μL of water was added to the solution before it was covered with a silicone cover. Further, it underwent vortex oscillation for 2 min; then, the 96-well plate was centrifuged at 4000 rpm and room temperature for 5 min. Lastly, the 96-well plate was placed into the automatic sampler for analysis.

### Detection Analysis

The samples were analyzed by a liquid chromatography I-Class Xevo system (Waters, America) coupled with mass spectrometry TQ-S IVD (Waters, America). The parameters used in this study are as follows: (1) the chromatographic column was C18 column; (2) the pre-heating temperature of the chromatographic column was 50°C; (3) the mobile phase A was H2O/A liquid=100/0.1; (4) the mobile phase B was MeOH/B liquid=100/0.1; (5) the chromatographic injection volume was 2 ul; (6) the ion source voltage was 5500 V; (7) the ionization source temperature was set to 450°C. Limit of quantification (LOQ) and internal standard (IS) for steroid hormones analyzed by LC-MS/MS were showed in **Table S1**.

Performance index of this method: The relative deviation of accuracy shall not exceed ± 15.0%, and the correlation coefficient (R) shall not be less than 0.9900. The repeatability (coefficient of variation, CV) shall not exceed 15.0%. Inter batch difference shall not exceed 20.0%.

### Statistical Analyses

All analyses were performed in R (version 3.6.3, http://www.R-project.org). Summary statistics for normally distributed variables were expressed as the mean ± standard deviation, and non-normally distributed quantitative variables were expressed as the median plus interquartile range. Categorical data were summarized as numbers and percentages. The Mann-Whitney U test or Kruskal-Wallis test was performed for comparisons between two or more groups. Differences were considered statistically significant at a two-sided p value of 0.05.

## Results

In this study, 18 different steroid hormone metabolites were experimented in the maternal-fetal interface, including five types of estrogens, four types of androgens, eight types of glucocorticoids, and one type of mineralocorticoid. Except for dihydrotestosterone in the placenta, most hormones have obtained effective results by MS. All the results are shown in **Tables 2**–**4**.

**Table 2:** Comparison of hormone levels in maternal blood (median (Q1~Q3), ng/ml).

Hormone	Control	AMA			P1	P2
		Total	AMA1	AMA2		
**Estrogens**						
Estrone (E1)	3.62 (2.46-4.70)	3.38 (2.40-5.61)	4.14 (2.13-5.38)	3.22 (3.00-5.70)	0.687	0.539
Estradiol (E2)	17.14 (13.10-22.25)	17.65 (13.47-21.89)	16.99 (13.39-21.68)	19.67 (14.57-24.35)	0.803	0.661
Estriol (E3)	10.31 (7.42-11.96)	12.08 (9.88-14.60)	12.08 (9.13-14.32)	12.28 (10.86-14.40)	0.140	0.792
Progesterone (P4)	125.19 (98.06-153.51)	118.42 (101.58-158.88)	115.75 (101.04-158.36)	137.48 (120.89-168.84)	0.715	0.483
17 α-hydroxyprogesterone(17α-OHP)	4.44 (3.68-7.70)	6.55 (5.60-7.06)	6.37 (4.98-7.13)	6.58 (6.06-6.73)	0.212	0.599
**Androgens**						
Testosterone (T)	1.36 (0.89-2.42)	0.88 (0.58-1.19)	0.68 (0.39-0.98)	1.55 (1.05-2.34)	**0.010**	**0.023**
Androstenedione (A2)	2.06 (1.37-3.56)	1.30 (0.73-1.69)	0.92 (0.69-1.31)	2.27 (1.52-3.27)	**0.007**	**0.011**
Dehydroepiandrosterone(DHEA)	1.82 (0.93-2.65)	1.73 (1.45-2.34)	1.50 (1.19-1.91)	2.39 (2.03-2.75)	0.471	**0.028**
Dihydrotestosterone (DHT)	0.19 (0.15-0.30)	0.12 (0.10-0.18)	0.11 (0.08-0.14)	0.18 (0.16-0.19)	**0.021**	0.066
**Glucocorticoid**						
Cortisol (F)	199.06 (166.80-259.24)	221.55 (173.73-289.77)	208.73 (168.47-282.10)	277.99 (233.72-312.87)	0.715	0.38
Cortisone (E)	43.68 (34.21-48.30)	50.37 (37.27-52.84)	40.43 (37.16-51.72)	50.71 (48.47-55.72)	0.659	0.38
Cortisol/Cortisone Ratio	5.06 (4.32-5.92)	5.25 (4.38-5.59)	4.94 (4.12-5.68)	5.29 (5.05-5.46)	0.659	0.43
Corticosterone (CC)	3.62 (2.87-5.55)	3.81 (2.68-5.18)	3.81 (2.69-5.16)	3.82 (2.37-5.05)	0.924	0.861
21-deoxycortisol (21-DOC)	0.07 (0.02-0.10)	0.06 (0.03-0.08)	0.06 (0.04-0.09)	0.02 (0.02-0.03)	1.000	0.186
11-deoxycortisol (11-DOC)	1.50 (1.00-2.37)	1.87 (1.17-2.64)	1.73 (1.14-2.80)	2.04 (1.74-2.42)	0.715	0.726
Pregnenolone (PREG)	1.69 (1.29-2.05)	2.26 (0.98-3.14)	2.30 (1.74-4.00)	1.28 (0.67-3.03)	0.145	0.462
17-OH Pregnenolone (17-OHP5)	1.25 (0.95-1.45)	1.17 (0.94-1.67)	1.10 (0.94-1.54)	1.89 (1.31-2.00)	0.803	0.054
Dexamethasone (DXMS)	0.01 (0.00-0.01)	0.01 (0.00-0.03)	0.01 (0.00-0.03)	(-)	0.652	
**Mineralocorticoid**						
Deoxycorticosterone (DOCA)	0.32 (0.21-0.46)	0.28 (0.22-0.39)	0.28 (0.23-0.43)	0.31 (0.23-0.35)	0.687	0.861

AMA1, without complications of pregnancy; AMA2, with complications of pregnancy. P1, Compare between AMA1 and control group; P2, Compare between AMA1 and AMA2; Bold values indicate P < 0.05.

**Table 3:** Comparison of hormone levels in placenta (median (Q1~Q3), ng/mg).

Hormone	Control	AMA			P1	P2
		Total	AMA1	AMA2		
**Estrogens**						
Estrone (E1)	11.33(6.64-21.28)	16.03(11.28-23.47)	14.65(9.93-20.79)	23.91(14.03-32.86)	0.536	0.138
Estradiol (E2)	1.00(0.80-2.21)	1.22(0.69-1.72)	1.28(0.72-2.25)	1.14(0.78-1.40)	0.799	0.458
Estriol (E3)	41.2(32.81-57.79)	44.5(41.16-67.36)	43.44(41.94-69.76)	52.84(40.90-61.93)	0.216	0.934
Progesterone (P4)	550.56(375.48-674.58)	573.27(473.08-660.98)	524.36(468.28-662.10)	604.49(572.10-648.39)	0.716	0.509
17 α-hydroxyprogesterone(17α-OHP)	4.04(2.90-6.03)	4.27(3.83-7.06)	4.27(3.51-5.82)	6.41(4.12-9.11)	0.536	0.509
**Androgens**						
Testosterone (T)	0.08(0.04-0.11)	0.1(0.03-0.13)	0.1(0.04-0.15)	0.09(0.05-0.11)	0.742	0.585
Androstenedione (A2)	0.76(0.42-1.04)	0.57(0.49-0.86)	0.57(0.50-0.79)	0.68(0.46-1.10)	0.597	0.805
Dehydroepiandrosterone(DHEA)	0.73(0.65-0.97)	0.84(0.71-0.99)	0.87(0.72-1.04)	0.84(0.73-0.89)	0.173	0.357
Dihydrotestosterone (DHT)	-	-	-	-		-
**Glucocorticoid**						
Cortisol (F)	0.22(0.10-0.60)	0.26(0.21-0.39)	0.26(0.22-0.37)	0.3(0.20-0.54)	0.597	0.564
Cortisone (E)	36.33(26.66-51.33)	38.78(24.46-49.21)	35.56(23.37-44.97)	43.99(40.58-68.00)	0.610	0.058
Cortisol/Cortisone Ratio	0.01(0.01-0.02)	0.01(0.01-0.02)	0.01(0.01-0.01)	0.01(0.01-0.02)	0.610	0.805
Corticosterone (CC)	0.07(0.03-0.12)	0.1(0.04-0.15)	0.05(0.02-0.13)	0.14(0.12-0.20)	0.851	0.054
21-deoxycortisol (21-DOC)	0(0.00-0.00)	0(0.00-0.00)	0(0.00-0.00)	0(0.00-0.00)	0.656	0.793
11-deoxycortisol (11-DOC)	0.8(0.60-1.28)	1.22(0.72-1.60)	1.22(0.79-1.56)	1.12(0.62-1.95)	0.190	0.934
Pregnenolone (PREG)	42.74(24.24-83.48)	57.32(32.27-79.16)	50.97(26.31-77.52)	67.91(57.84-95.23)	0.971	0.248
17-OH Pregnenolone (17-OHP5)	0.5(0.29-0.71)	0.59(0.43-0.68)	0.56(0.44-0.61)	0.71(0.46-0.85)	0.610	0.322
Dexamethasone (DXMS)	-	-	-	-		
**Mineralocorticoid**						
Deoxycorticosterone (DOCA)	0.98(0.54-1.53)	1.01(0.85-1.36)	0.99(0.80-1.33)	1.11(0.97-1.34)	0.662	0.536

AMA1, without complications of pregnancy; AMA2, with complications of pregnancy. P1, Compare between AMA1 and control group; P2, Compare between AMA1 and AMA2.

**Table 4:** Comparison of hormone levels in cord blood (median (Q1~Q3), ng/ml).

Hormone	Control	AMA			P1	P2
		Total	AMA1	AMA2		
**Estrogens**						
Estrone (E1)	9.75 (7.17-19.26)	11.53 (8.09-16.64)	9.45 (6.84-12.31)	14.67 (11.93-18.37)	0.595	**0.048**
Estradiol (E2)	2.86 (2.20-4.51)	2.83 (2.10-3.90)	2.83 (2.14-3.16)	4.95 (2.24-8.79)	0.494	0.322
Estriol (E3)	82.14 (67.34-127.87)	97.42 (76.88-139.18)	81.90 (74.60-120.31)	121.83 (110.22-160.27)	0.970	0.058
Progesterone (P4)	443.26 (339.37-555.52)	391.52 (308.62-516.39)	354.39 (276.98-484.16)	452.38 (379.38-525.80)	0.210	0.284
17 α-hydroxyprogesterone(17α-OHP)	10.14 (8.75-13.36)	9.96 (7.67-14.73)	9.45 (6.99-11.06)	13.17 (10.10-20.24)	0.649	0.248
**Androgens**						
Testosterone (T)	0.10 (0.08-0.11)	0.10 (0.08-0.22)	0.13 (0.08-0.23)	0.09 (0.07-0.18)	0.193	0.43
Androstenedione (A2)	0.36 (0.32-0.44)	0.38 (0.31-0.50)	0.37 (0.30-0.49)	0.41 (0.36-0.49)	0.690	0.458
Dehydroepiandrosterone(DHEA)	0.64 (0.35-0.69)	0.69 (0.51-0.87)	0.69 (0.48-0.99)	0.66 (0.55-0.79)	0.271	1
Dihydrotestosterone (DHT)	0.10 (0.10-0.11)	0.11 (0.10-0.11)	0.10 (0.10-0.12)	0.11 (0.11-0.11)	0.689	0.699
**Glucocorticoid**						
Cortisol (F)	14.19 (11.20-19.28)	18.11 (12.03-23.36)	18.67 (13.35-26.02)	13.09 (11.68-18.47)	0.254	0.509
Cortisone (E)	75.35 (67.91-91.31)	75.30 (60.53-89.18)	64.24 (59.51-86.29)	77.40 (76.68-99.10)	0.323	0.216
Cortisol/Cortisone Ratio	0.18 (0.13-0.28)	0.26 (0.19-0.32)	0.28 (0.20-0.40)	0.21 (0.14-0.27)	0.074	0.187
Corticosterone (CC)	0.71 (0.49-0.90)	0.70 (0.45-0.85)	0.52 (0.36-0.78)	0.87 (0.73-1.00)	0.173	0.068
21-deoxycortisol (21-DOC)	0.02 (0.01-0.04)	0.01 (0.01-0.02)	0.01 (0.01-0.02)	0.01 (0.01-0.01)	0.269	0.373
11-deoxycortisol (11-DOC)	2.13 (1.83-2.57)	2.46 (2.21-4.05)	2.44 (1.81-3.41)	3.23 (2.38-4.14)	0.569	0.284
Pregnenolone (PREG)	6.47 (5.71-7.38)	6.11 (5.21-8.87)	5.62 (4.88-7.88)	8.95 (6.42-9.61)	0.569	0.216
17-OH Pregnenolone (17-OHP5)	2.36 (1.70-2.84)	2.88 (2.01-3.84)	2.88 (2.04-3.62)	3.19 (1.98-4.32)	0.254	0.741
Dexamethasone (DXMS)	0.07 (0.07-23.59)	0.07 (0.06-0.07)	0.07 (0.07-0.07)	0.06 (0.06-0.07)	0.319	0.158
**Mineralocorticoid**						
Deoxycorticosterone (DOCA)	0.53 (0.48-0.69)	0.57 (0.42-0.80)	0.45 (0.40-0.74)	0.74 (0.56-0.94)	0.279	0.099

AMA1, without complications of pregnancy; AMA2, with complications of pregnancy. P1, Compare between AMA1 and control group; P2, Compare between AMA1 and AMA2; Bold values indicate P < 0.05.

### Profiles of Steroid Hormone in Maternal-Fetal Interface

**Figure 1:**
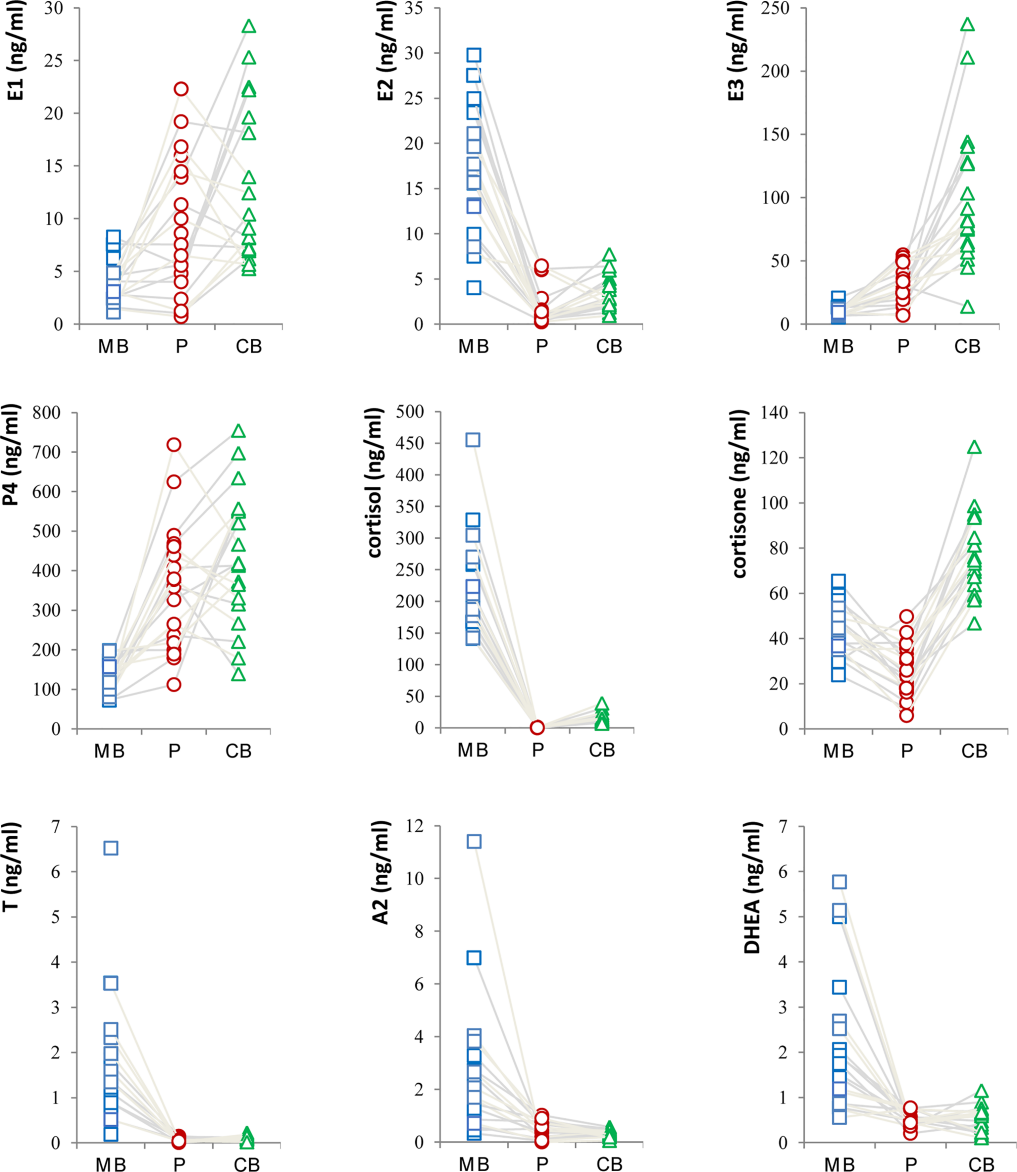
Profiles of Steroid Hormone in Maternal-Fetal Interface of normal pregnant women.

In this study, the levels of major steroid hormones in maternal and fetal samples of the control group were compared in **Figure 1**. The levels of E2 in MB were higher than those in P and CB samples. In contrast, E1 and E3 levels had similar changes and were higher in P and CB. Compared with MB, P4 was higher in P and CB. In addition, the levels of cortisol (F) were deficient in P and CB. In contrast, its levels in MB were maintained at an elevated level. However, the levels of cortisone (E) in CB were higher than those in MB and P. Furthermore, three main androgens [Testosterone (T), Androstenedione (A2), and Dehydroepiandrosterone (DHEA)] in the maternal-fetal interface showed a similar state. Maternal androgens were higher than the other two. They were deficient in P and CB. Relatively, DHEA in CB was slightly higher than in P.

### Cascade Changes of Steroid Hormones

**Figure 2:**
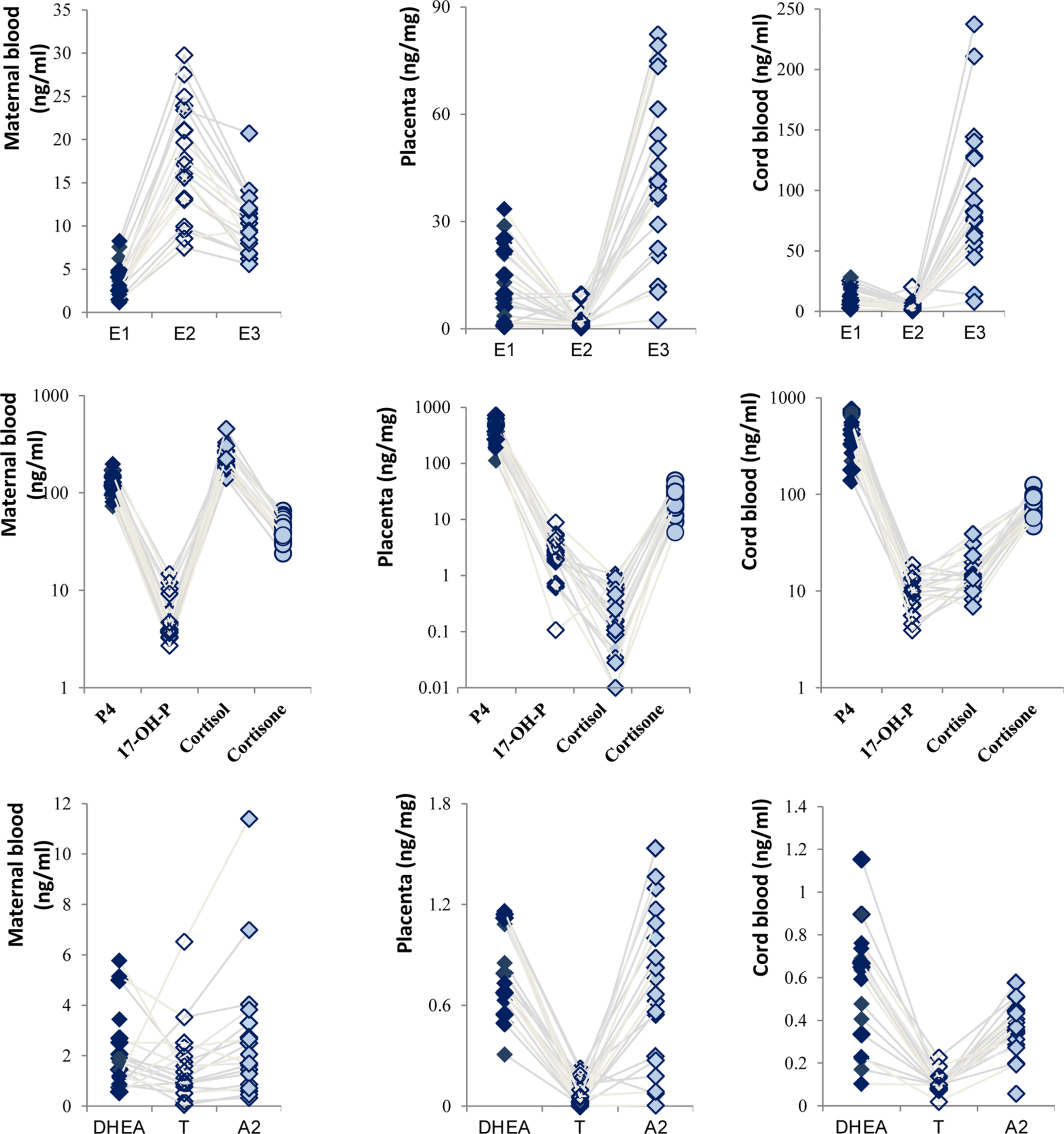
Cascade changes of steroid hormones in normal pregnant women.

Steroid hormones are derived from cholesterol and produced by cascade reactions of enzymes, including the cytochrome P450 enzyme and hydroxysteroid dehydrogenase. Still based on the control group, the estrogen displayed an inverted V-shaped relationship in MB due to having the highest concentration of E2 (**Figure 2**). On the other hand, a V-shaped relationship was presented in P and CB. Meanwhile, E3 levels were higher than others in CB, and the levels of P4 in P and CB were much higher than those of cortisol and cortisone. Our results showed that placental P4 was 15-fold higher than cortisone in ordinary pregnant women and 5.88-fold in CB. Cortisone levels were higher than cortisol levels in P and CB. In contrast, in maternal cortisol levels were higher than P4 and cortisone levels. Furthermore, the relationship of T, A2, and DHEA was consistent. T was at a low level.

### Steroid Profiles of Pregnant Women at Advanced Maternal Age

To our surprise, except for T, A2 and Dihydrotestosterone (DHT), there were no significant differences in the other 15 steroid hormones in MB between the AMA1 and the control group (p>0.05). To the simple AMA women, who didn’t occur adverse pregnancy complications, the levels of these three androgens (T, A2 and DHT) significantly reduced (p<0.05, **Table 2**). Subsequently, compared with the AMA1 group, androgen levels were significantly higher in AMA2, especially in T (1.55 *vs.* 0.68 ng/ml, p=0.023), A2 (2.27 *vs.* 0.92 ng/ml, p=0.011) and Dehydroepiandrosterone (DHEA) (2.39 *vs.* 1.50 ng/ml, p=0.028). In placenta, there were no significant changes in any of the steroid levels between AMA and controls (p>0.05, **Table 3**). Similarly, whether AMA women had adverse pregnancy complications or not, it had no relationship with placental steroids. Likewise, compared with normal pregnant women, most of the steroid hormone levels in CB of AMA women did not significantly change (p>0.05, **Table 4**). However, E1 was significantly increased in AMA women with adverse pregnancy complications (14.67 *vs.* 9.45 ng/ml, p=0.048).

Moreover, among 20 ordinary women, there were 9 boys and 11 girls after birth. The relationship between neonatal sex and these steroid hormones was further compared. As shown in **Table S2**, the only significant difference was on the A2 levels in CB. The boy’s A2 level significantly increased (0.12 *vs.* 0.08 ng/ml, p<0.001).

## Discussion

As we all know, AMA pregnant women are at considerable risk of adverse pregnancy complications and poor fetal outcomes. Nevertheless, we still cannot explain the reason clearly. Recently, increasing attention has been made to the vital role of steroid hormones during pregnancy, but the studies are still minimal. In this preliminary study, we explored the status of major steroid hormones in the maternal-fetal interface of AMA pregnant women for the first time. Our results suggested certain distribution rules and cascade changes of steroid profiles in the maternal-fetal compartments. This can contribute to understand their biosynthesis and transmission during pregnancy further. At the same time, we preliminarily revealed the characteristic changes of steroid hormone levels of AMA women in the third trimester. Although most hormones did not considerable change, the relationship between adverse pregnancy complications and significantly increased androgen in AMA women deserved our attention. It may also help us reveal the reasons for the high pregnancy risk of AMA pregnant women.

During pregnancy, there is a complex network of steroid synthesis and transmission in the maternal-fetal compartments (8, 9). A rule of synthesis and transport of steroid hormones exist between the mother, placenta, and fetus. Once the balance is broken, it will lead to adverse pregnancy and poor fetal outcomes. Vuppaladhadiam’s group (10) detected 10 types of steroid metabolites in P and CB using ultra-pure liquid chromatography (UPLC) MS, and they described a schematic of steroidogenesis in the maternal-fetal compartments. This diagram intuitively showed the synthesis, transport, and regulation of steroid hormones in the maternal-fetal interface (**Supplementary Figure 1**). It can help us have a clear preliminary understanding of this complex network. Inspired by their study, we analyzed 18 steroids in MB, P, and CB by similar techniques in a larger clinical sample. Our results confirmed Vuppaladhadiam’s report again and clarified the distribution characteristics of estrogen and glucocorticoid. Remarkably, compared with MB, P4 was higher in P and CB. We found placental P4 was 15-fold higher than cortisone in ordinary pregnant women and 5.88-fold in CB. Similarly, Vuppaladhadiam’s group also reported that placental P4 was ~13-fold higher than cortisone levels (10). These results support the theory that the placenta is the primary source of P4 during pregnancy (11). Cortisol and cortisone also play a vital role in pregnancy (12). The high concentration of cortisol from the mother needs to be rapidly converted to its inactive metabolite (cortisone) under the actions of the enzyme hydroxysteroid 11β dehydrogenase type 2 (13). This enzyme is the key to protecting the fetus’s normal growth. As expected, cortisol levels in MB were maintained at an elevated level while deficient in P and CB.

On the other hand, we expanded to more types of hormones, especially the distribution of androgens. There were few studies focused on androgen levels in P and the differences in their synthesis and secretion (14). The results of our study are consistent with those in Yoshida’s report (15), which also used the MS. Since the maternal androgen comes from the organs, except the placenta, the maternal androgen levels were slightly higher. The fetus’s level in CB is slightly higher than that in P because it is the main site for DHEA synthesis. However, compared with the other steroids, the concentrations of androgen were lower. It will be more difficult to draw the clinical conclusion because of the high technical requirements, which will avoid substantial individual differences, needed for detection.

With the increasing age of women > 30 years, ovarian activity and hormone production (mainly E2) will be reduced (16). On the other hand, due to the influence of placental endocrine function, there are substantial changes in pregnant women’s hormones (17). So, is there a profound change in steroid hormones in pregnant AMA women? Until now, there has been a lack of studies on steroids in AMA pregnant women. Plant M et al. (18) reported a negative association between maternal age and circulating E2, P4, and levels in the third trimester. They used thirteen age-diverse pregnant vervet monkeys as study patients, which were considered a preclinical model to study the effects of AMA on adaptations to pregnancy. Only four monkeys were part of the AMA group. In this study, we conducted one clinical study from three types of maternal-fetal samples. However, our results did not seem to agree with Plant’s. Except for the decline of T, A2 and DHT, there were no significant differences in the other 15 steroid hormones in MB between AMA1 and the control group. Subsequently, the levels of androgens significantly increased in the AMA women who had adverse pregnancy complications. This may relate to the objects and methods used in the research.

In the past, many studies focused on the roles of steroids during pregnancy, including P4, estrogen, cortisol, cortisone and others, but studies including androgens were relatively limited. Androgens are known to secrete at a woman’s ovaries, adrenal glands, and placenta, the main source during pregnancy. Recent studies reported that androgen levels of pregnant women increased in maternal plasma compared with those in non-pregnant women (19), as precursors for E2 synthesis, DHEA and T, play essential roles in pregnancy (14). It is particularly noteworthy that androgen levels increase further when women have pregnancy complications related to placental dysfunction. For example, androgens are considered a contributor to the occurrence and development of PE. In late pregnancy, maternal plasma T levels of PE were 1.5 to 2.4-fold higher than those in normal pregnant women (20). In present study, we found an interesting result that when the AMA women didn’t occurred adverse pregnancy complications, the level of T, A2 and DHT significantly reduced. However, these androgens would significantly increase when they combined with adverse pregnancy. Most notably, the levels of T, A2, and DHEA in AMA2 women were 2.27-fold, 2.46-fold, and 1.59-fold higher than the AMA1 group. It suggests that abnormally increased androgens may be an essential factor in the considerable risk of AMA women. This may open a new field for us. Can the regulation of steroid hormones reduce the pregnancy risk of elderly pregnant women? Of course, but the current study is still superficial and needs more in-depth basic and clinical research.

Nevertheless, the present preliminary study has some limitations. First, it is still an initial observational and descriptive clinical study. No exploration of relevant molecular mechanisms has been conducted. Patients were recruited in an unstructured way, and this may have resulted in non-representative groups. Due to the lack of sample size, this study did not conduct a comparative analysis about different age stages, relationship with pregnancy outcomes, and so on. In addition, the age difference between the AMA group and control group is little, so the power to detect changes with age is somewhat low. The interpretation of the results is speculative and requires further verification. The tandem MS technology needs to be further improved, especially for P.

To conclude, we explored the status of major steroid hormones in the maternal-fetal interface of AMA pregnant women for the first time. Our results revealed the distribution rules and cascade changes of steroid profiles in maternal–fetal compartments. There is a positive relationship between adverse pregnancy complications and significantly increased androgen in AMA women. It may help us reveal the reasons for the high pregnancy risk of AMA pregnant women.

## Data Availability Statement

The original contributions presented in the study are included in the article/**Supplementary Material**. Further inquiries can be directed to the corresponding authors.

## Ethics Statement

The study design and protocol were reviewed and approved by the ethics committee of Changzhou Maternal and Child Health Care Hospital. Informed consents were obtained from all individual participants included in the study. The patients/participants provided their written informed consent to participate in this study.

## Author Contributions

BY, FG, and JZ carried out the assays and participated in the study design. BY, FG, YY, and JZ carried out clinical consultations and laboratory tests. WL performed the statistical analysis. BY and JZ conceived the study, participated in its design and coordination and helped draft the manuscript. All authors contributed to the article and approved the submitted version.

## Funding

This study was funded by Project supported by National Natural Science Foundation of China (81773438), Changzhou Key Laboratory of High-tech Research (CM20193009) and Jiangsu Maternal and Children Health Care Key Discipline (FXK201754).

## Conflict of Interest

The authors declare that the research was conducted in the absence of any commercial or financial relationships that could be construed as a potential conflict of interest.

## Publisher’s Note

All claims expressed in this article are solely those of the authors and do not necessarily represent those of their affiliated organizations, or those of the publisher, the editors and the reviewers. Any product that may be evaluated in this article, or claim that may be made by its manufacturer, is not guaranteed or endorsed by the publisher.
