# Supplementary material for: Steroidomics of Pregnant Women at Advanced Age
Source: Front Endocrinol (Lausanne). 2022 Feb 23;13:796909. doi: 10.3389/fendo.2022.796909 (PMC8905515; doi:10.3389/fendo.2022.796909)
Supplement: Supplementary file 1 [file DataSheet_1.doc]

**Supplementary figure 1.** **Schematic of steroidogenesis in maternal–fetal compartments during pregnancy.**

**
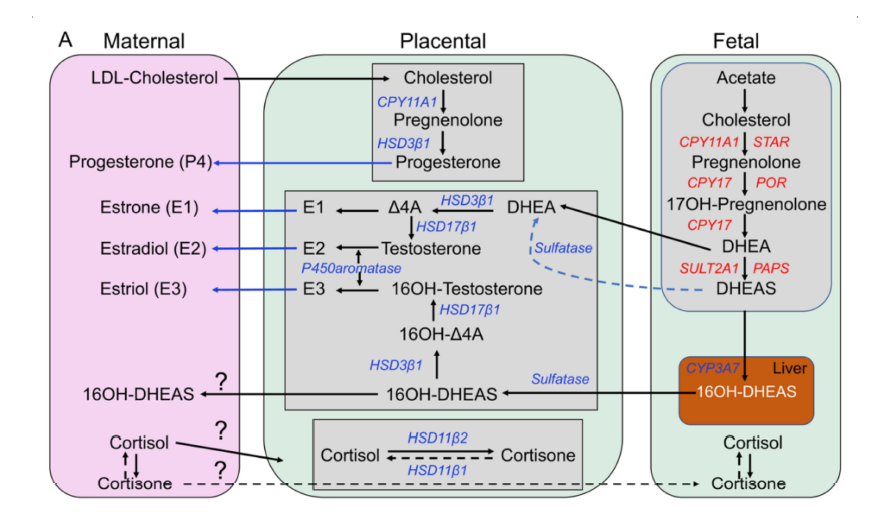
**

Note: Maternal cholesterol is transported to the placenta, where it serves as a precursor for the production of progesterone and its metabolites. Maternal glucocorticoids can be transported to the placenta and across to the fetal circulation. The fetus makes its own cholesterol from acetate and converts it to DHEAS via several steps, as shown. DHEAS is transported to the fetal liver and from there to the placenta, where DHEAS is converted to testosterone and ultimately to estrogens (estrone (E1), estradiol (E2), and estriol (E3)), which are subsequently transported to the maternal circulation from the placenta.

Cite from: Vuppaladhadiam L, Lager J, Fiehn O, Weiss S, Chesney M, Hasdemir B, Bhargava A. Human Placenta Buffers the Fetus from Adverse Effects of Perceived Maternal Stress. Cells. 2021 Feb 12;10(2):379. doi: 10.3390/cells10020379. PMID: 33673157; PMCID: PMC7918582.
