# Supplementary material for: Steroidomics of Pregnant Women at Advanced Age
Source: Front Endocrinol (Lausanne). 2022 Feb 23;13:796909. doi: 10.3389/fendo.2022.796909 (PMC8905515; doi:10.3389/fendo.2022.796909)
Supplement: Supplementary file 2 [file DataSheet_2.docx]

**Table S1: Serum LOQ and internal standard for steroid hormones analyzed by LC-MS/MS**

| **Steroid hormone** | **LOQ（ng/ml）** | **IS** |
| --- | --- | --- |
| Dehydroepiandrosterone | 0.5 | DHEA-d6 |
| Androstenedione | 0.01 | A4-d7 |
| testosterone | 0.05 | -- |
| Dihydrotestosterone | 0.1 | DHT-d4 |
| Pregnenolone | 0.1 | Preg-d4 |
| progesterone | 0.3 | P-d9 |
| Deoxycorticosterone | 0.02 | DOC-d8 |
| 17 α-hydroxyprogesterone | 0.05 | 17α-OH P-d8 |
| corticosterone | 0.05 | -- |
| 21-deoxycortisol | 0.02 | -- |
| 11deoxycortisol | 0.05 | 11-Deo-d7 |
| Cortisone | 0.05 | Cortisone-d7 |
| cortisol | 0.2 | Cortisol-d4 |
| Dexamethasone | 0.5 | Dexamethasone-d4 |
| Estrone | 0.1 | E1-d4 |
| Estradiol | 0.2 | E2-d4 |
| Estriol | 0.2 | E3-d4 |
| 17-hydroxypregnenolone | 0.2 | -- |

LOQ: Limit of quantification; IS：Internal standard

**Table S2. Comparison of hormone levels in normal pregnant women by fetal sex (median (Q1~Q3))**

| **Hormone** | **Male** | **Female** | **P** |
| --- | --- | --- | --- |
| **Number** | 9 | 11 |  |
| **Maternal blood** | | | |
| E1 | 2.53 (1.59-4.06) | 4.14 (2.88-4.76) | 0.221 |
| E2 | 15.58 (9.49-19.64) | 19.08 (15.77-22.83) | 0.327 |
| E3 | 9.50 (6.78-12.07) | 10.56 (8.31-11.33) | 0.744 |
| P4 | 119.73 (95.63-125.19) | 146.88 (107.05-167.60) | 0.221 |
| T | 1.59 (0.95-2.33) | 1.23 (0.80-2.32) | 0.568 |
| A2 | 2.66 (1.63-3.83) | 1.88 (1.01-2.67) | 0.514 |
| DHEA | 1.82 (0.84-2.88) | 1.72 (1.14-2.41) | 0.79 |
| CRTL | 211.63 (177.27-269.78) | 193.17 (165.40-221.24) | 0.414 |
| CRTN | 44.62 (35.02-49.20) | 41.59 (34.23-47.36) | 0.87 |
| Placenta | | | |
| E1 | 9.77(7.24-17.01) | 12.14(6.63-24.15) | 0.462 |
| E2 | 0.95(0.86-9.03) | 1.5(0.79-1.93) | 0.806 |
| E3 | 41.2(36.44-54.13) | 39.43(26.11-57.47) | 0.806 |
| P4 | 537.96(397.51-569.17) | 580.46(387.42-682.81) | 0.744 |
| T | 0.05(0.04-0.09) | 0.1(0.05-0.12) | 0.366 |
| A2 | 0.67(0.56-0.88) | 0.79(0.36-1.15) | 0.744 |
| DHEA | 0.67(0.63-1.12) | 0.76(0.68-0.84) | 0.683 |
| CRTL | 0.18(0.16-0.70) | 0.23(0.04-0.43) | 0.624 |
| CRTN | 46.76(27.90-53.38) | 33.12(26.46-42.36) | 0.369 |
| **Cord blood** |  |  |  |
| E1 | 8.18 (6.19-12.44) | 13.96 (9.09-22.50) | 0.07 |
| E2 | 2.79 (2.10-2.92) | 4.28 (2.33-5.03) | 0.2 |
| E3 | 77.81 (74.90-103.52) | 82.72 (62.37-144.24) | 0.566 |
| P4 | 466.62 (315.48-550.16) | 419.91 (365.13-557.33) | 0.627 |
| T | 10.26 (8.52-12.92) | 10.02 (9.59-13.62) | 0.566 |
| A2 | 0.12 (0.10-0.14) | 0.08 (0.08-0.08) | **<0.001** |
| DHEA | 0.35 (0.29-0.41) | 0.39 (0.35-0.45) | 0.31 |
| CRTL | 0.67 (0.41-0.74) | 0.59 (0.34-0.65) | 0.508 |
| CRTN | 0.10 (0.10-0.12) | 0.10 (0.09-0.10) | 0.462 |
